# The incidence of eosinophilic oesophagitis in 2007–2017 among children in North Denmark Region is lower than expected

**DOI:** 10.1186/s12887-022-03258-6

**Published:** 2022-04-06

**Authors:** Martin Hollænder, Jacob Holmen Terkelsen, Frederik Kramme, Kasper Bredal, Kristian Kragholm, Kasper Dalby, Søren Hagstrøm, Dorte Melgaard, Anne Lund Krarup

**Affiliations:** 1 School of Medicine and Health, Aalborg University, Frederik Bajers Vej 7D, 9220 Aalborg, Denmark; 2 Department of Cardiology, Unit of Clinical Biostatistics and Epidemiology, Aalborg University Hospital, Hobrovej 18-22, 9100 Aalborg, Denmark; 3 Pediatric Center, Slotsgade 20C, 5000 Odense, Denmark; 4 Department of Pediatrics, Aalborg University Hospital, Hobrovej 18-22, 9100 Aalborg, Denmark; 5 Center for Clinical Research, North Denmark Regional Hospital, Bispensgade 37, 9800 Hjørring, Denmark; 6 Department of Clinical Medicine, Aalborg University, Frederik Bajers Vej 7D, 9220 Aalborg, Denmark; 7 Department of Acute Medicine and Trauma Care, Aalborg University Hospital, Hobrovej 18-20, 9000 Aalborg, Denmark; 8 Department of Gastroenterology, Aalborg University Hospital, Hobrovej 18-22, 9100 Aalborg, Denmark

**Keywords:** Eosinophilic oesophagitis, Incidence, Child, Eosinophilia, Oesophagitis, Treatment

## Abstract

**Background:**

In North Denmark Region (NDR), the incidence of Eosinophilic Oesophagitis (EoE) among adults has increased following a new biopsy protocol in 2011, whereas data on the incidence of EoE among children is lacking.

**Aims:**

To describe the incidence of EoE in children aged 0–17 in NDR as well as diagnostic delay, clinical manifestations, treatment and complications.

**Methods:**

This retrospective, register-based DanEoE cohort study included 18 children diagnosed with EoE between 2007–2017 in NDR. Medical files were reviewed with attention to symptoms, reason for referral, disease progress, treatment, symptomatic and histological remission as well as diagnostic delay.

**Results:**

The median incidence per year (2007–2017) was 0.86/100,000 children in NDR aged 0–17 years. The median diagnostic delay among children was four years and six months. Sixty percent presented with food impaction at first hospital visit. After initial treatment, only one of 18 children achieved symptomatic and histologic remission and had a long-term treatment plan.

**Conclusions:**

The calculated incidence among children was lower compared to similar studies. Combined with poor remission rates and lack of follow-up, it is likely that EoE is an underdiagnosed and insufficiently treated disease among children in NDR. Our findings suggest that more knowledge concerning EoE in children could lead to a higher incidence, shorter diagnostic delay and more effective treatment.

## Introduction

Internationally, eosinophilic oesophagitis (EoE) is defined as a distinct clinicopathologic disease [1]. It is a chronic, immune/antigen-mediated oesophageal disease characterized by oesophageal dysfunction and eosinophil infiltration of the oesophageal epithelium (> 15 eosinophils per high-power field (EOS/HPF) [2, 3]. Adults with EoE most often present with dysphagia and food impaction, whereas clinical manifestations among children are less specific and vary with age. Infants and toddlers may suffer from food refusal and failure to thrive, school-aged children from vomiting or pain located to the abdomen, chest, or throat and adolescents may display food avoidance and clinical manifestations may resemble that of adults [3, 4]. EoE symptoms in children thus differ from that of adults, which challenges the diagnostic process in children. Moreover, literature shows that EoE is associated with considerable diagnostic delay [5]. If untreated, EoE can lead to irreversible structural changes such as fibrosis, strictures and dysfunction of the oesophagus [6]. These changes may lead to reduced oesophageal distensibility, which is associated with a higher risk of food impaction [7].

In recent years, the incidence of EoE has been increasing in the western world [8]. In a geographical region in Denmark, North Denmark Region (NDR), the incidence of EoE among adults has increased 50-fold to 6–8/100,000 following implementation of a new biopsy protocol in 2011. This increase suggests a previous lack of attention to, or knowledge of, the disease [9]. For children, one study from 2009 found the incidence of EoE in Region of Southern Denmark to be 1.6/100,000 [10], which is low compared to other countries comparable to Denmark [8].

The aim of this study was to describe EoE incidence, diagnostic delay, clinical manifestations, treatment and complications in children aged 0–17 in NDR.

## Methods

### Study population

This retrospective study used the DanEoE cohort. DanEoE is a database including oesophageal biopsy results of all patients in NDR between 2007–2017 with SNOMED codes for both oesophagus mucosa (T62010) and > 15 EOS/HPF (M47150). All medical files, endoscopy, radiology and histology reports, biochemistry results and referral documents until 31st December 2017 were reviewed in detail by an exprienced gastroenterologist and a gastroenterologist in training; subsequently, data were entered into the DanEoE database [11]. Details of DanEoE have been presented previously [9]. All management of data were performed in accordance with relevant guidelines and regulations.

In Denmark, all citizens have a unique civil registration number, which ensures complete linkage of data across national population-based medical registers as well as, including medical records, diagnostic codes, microbiologic, biochemistry, and pathologic findings [12, 13].

### DanEoE children

Data on children under 18 years of age with > 15 EOS/HPF in the oesophagus were retrieved from the DanEoE database. One child suffered from a disease possibly causing EoE-like symptoms. However, since EoE was suspected and symptoms as well as > 15 EOS/HPF in the oesophagus were present, the child was included in the study cohort. The medical files of the included children were reviewed by the authors of this study with attention to symptoms, reason for referral, disease progress, treatment, symptomatic and histological remission as well as diagnostic delay. Diagnostic delay is defined as the time between first sign or symptom of EoE and diagnosis of EoE. Second biopsy delay is the time from initial biopsy to second biopsy. Symptomatic remission delay is the time from initial treatment to symptomatic remission and histologic remission delay is the time from initial treatment to first oesophageal biopsy showing histologic remission (< 15 EOS/HPF). Findings were discussed with an experienced gastroenterologist before inclusion in the analysis. Children were divided into three age groups: 0–2, 3–12 and 13–17 years of age as symptoms present differently at different ages [3].

### Statistics

For descriptive statistics median and 1^st^ to 3^rd^ quartiles (Q1-Q3) were used for continuous variables and counts and percentages for categorical variables. Incidence of our study population was calculated on the basis of data from Statistics Denmark. The DanEoE database contains data from 2007 to 2017. Due to structural changes in 2008, the number of inhabitants in the North Region of Denmark before 2008 cannot be obtained. Instead, the number of inhabitants in 2008 was used to calculate the incidence in 2007. This was deemed justified as fluctutations in population size in NDR are relatively low. Regarding diagnostic delay, four children were excluded due to incomplete or non-accessible data in the medical files. The SAS enterprise guide 71 (SAS institute inc., Cary, North Carolina, USA) was used for data management and statistical analysis.

## Results

### Incidence

**Fig. 1:**
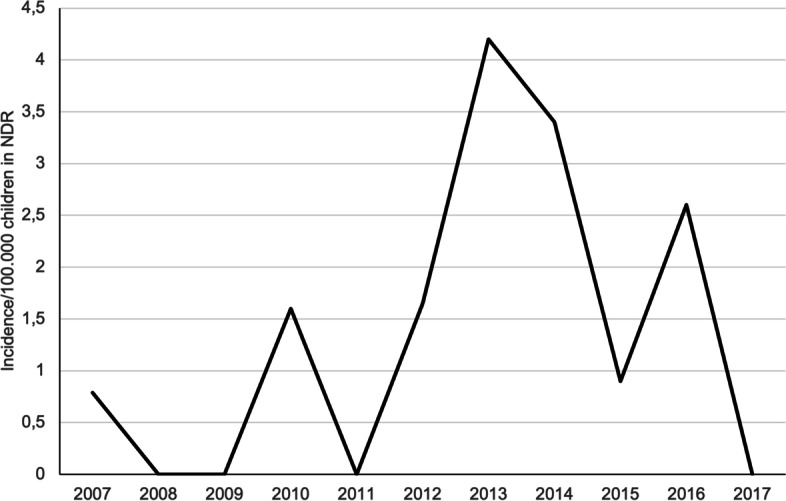
Incidence of Eosinophilic Oesophagitis in children aged 0–17 in North Denmark Region in the period 2007–2017

A total of 18 children were diagnosed with EoE in the period 2007–2017 (1 in 2007, 2 in 2009, 2 in 2012, 5 in 2013, 4 in 2014, 1 in 2015, 3 in 2016). In the years of 2008, 2009, 2011, and 2017 no children were diagnosed with EoE in NDR. The median incidence per year (2007–2017) was 0.86/100,000 (Fig. 1). No children aged 0–2 years with EoE were found; no children below the age of four years were diagnosed with EoE in our study population.

Frequency of upper endoscopy and biopsy on any indication.

**Fig. 2:**
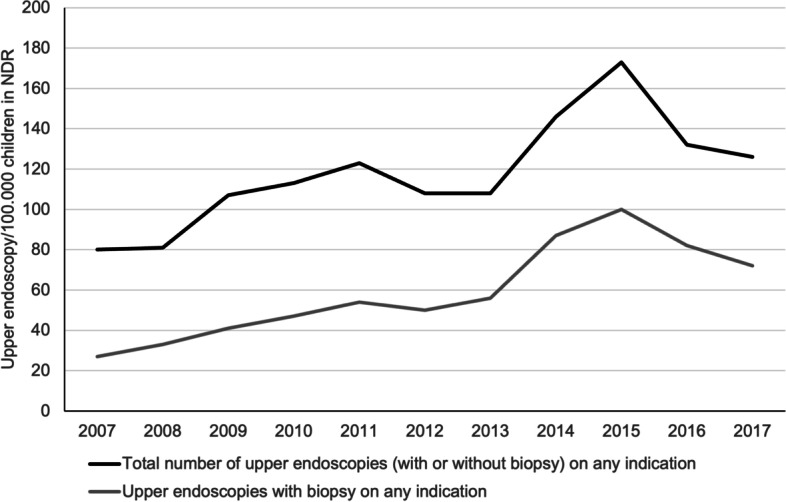
Upper endoscopies in children in NDR (2007–2017) on any indication. The total number of endoscopies was obtained from the regional registry using the procedures codes for “upper endoscopies without biopsies”, “upper endoscopies with biopsies”, and age groups < 18 years

The total number of upper endoscopies (black line) in children, and the subgroup of those endoscopies where biopsies were obtained (gray line) in NDR in the period 2007–2017 is shown in Fig. 2. No indications were omitted.

### Common clinical manifestations at first EoE-related hospital visit

**Fig. 3:**
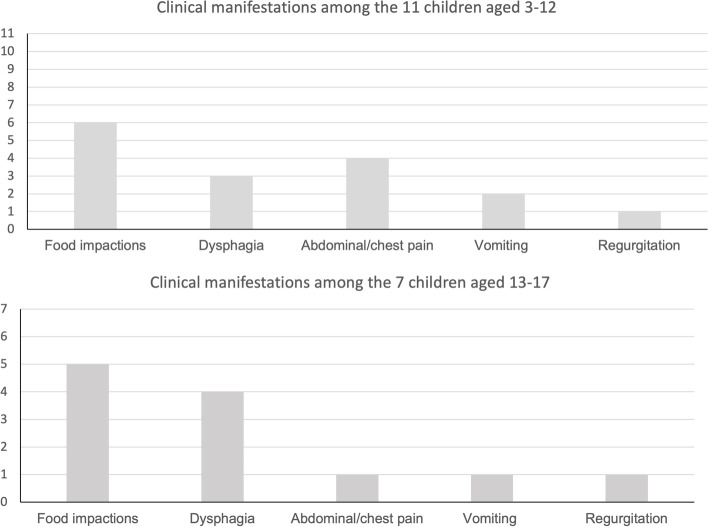
Clinical manifestations among children at their first hospital visit related to Eosinophilic Oesophagitis. School-aged children were defined as children aged 3–12, and adolescents were children aged 13–17. Remarkably, no infants or toddlers aged 0–2 were diagnosed in the period 2007–2017

The first EoE-related hospital visit in children aged 3–17 years were dominated by complaints of food impaction (Fig. 3). No children presented with signs of food avoidance; no children presented with failure to thrive or food refusal. The youngest child in our study population was three years and six months old at first EoE-related hospital visit.

At index endoscopy, two thirds of children had normal macroscopic findings (Table 1). A minority had macroscopic EoE signs. Edema and stenosis were not reported in our study population. Two children had other macroscopic findings: one had both ulcus and Mallory Weiss lesions, and one had food impaction. Oesophagitis was defined according to the LA classification [14]. In the endoscopy report of the patient with an ulcer, no LA-grade was reported.

**Table 1:** Study population demographics and macroscopic findings at index endoscopy

Number, (male/female)	18 (17/1)
Incidence (2007–2017)	0.86/100,000
Age at first EoE-related hospital visit, median years (Q1-Q3)	12 (11–14)
Age at first EoE-related hospital visit, n (%)	
0–2	0 (0%)
3–12	11 (61%)
13–17	7 (39%)
Macroscopic findings at index endoscopy, n (%)	
Macroscopic normal findings	12 (67%)
Macroscopic EoE findings, any type	4 (22%)
o Rings	2
o Furrows	2
o Edema	0
o White dots	1
o Stenosis	0
Scope laceration	0
Barretts oesophagus	0
Oesophagitis	0
Other macroscopic findings	2 (11%)

*Abbreviations*: *EoE* Eosinophilic Oesophagitis, *Q1* 1^st^ Quartile, *Q3* 3^rd^ quartile, *n* Number

### Diagnostic delay

**Fig. 4:**
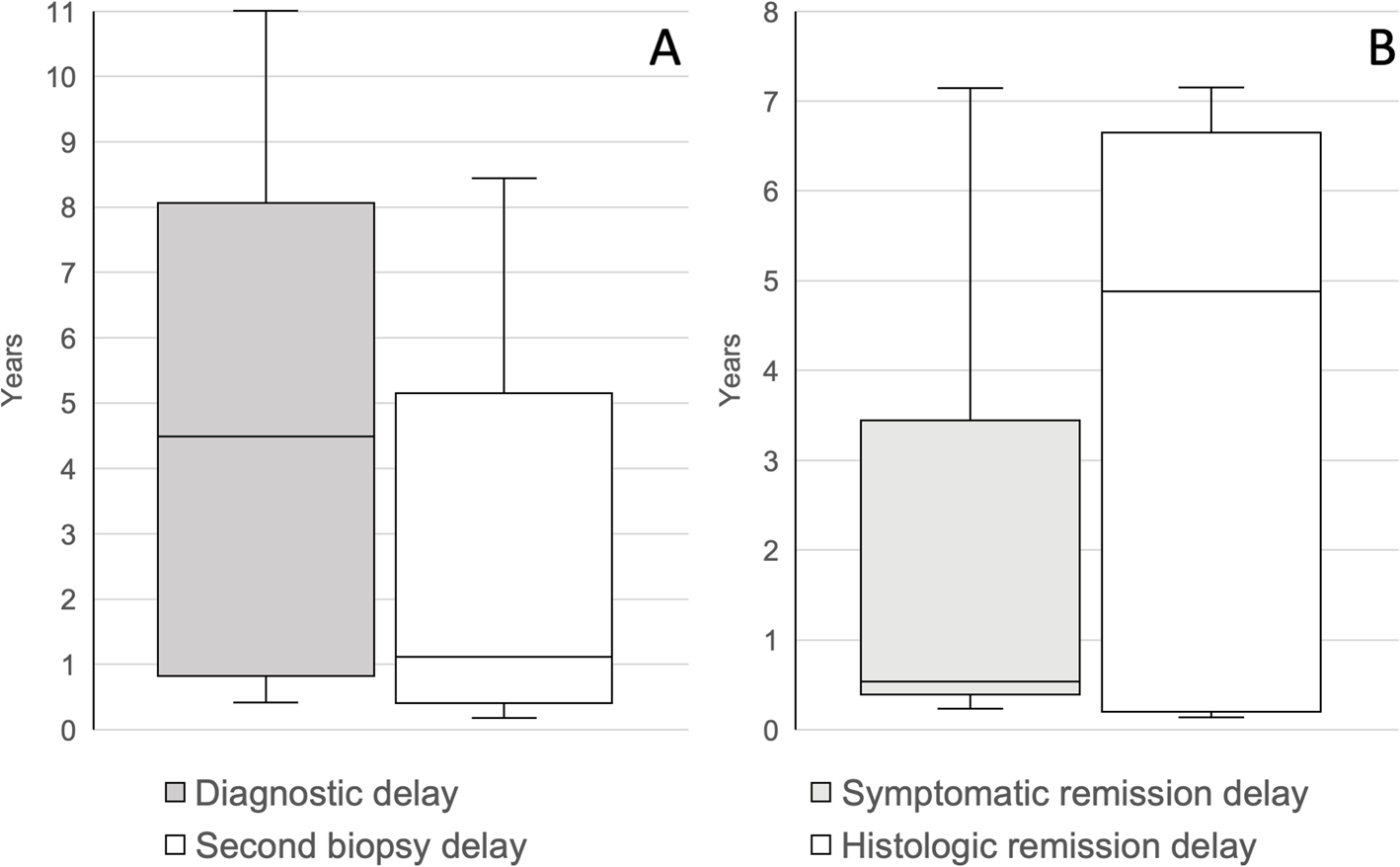
Diagnostic and second biopsy delay, and symptomatic and histologic remission delay in children with Eosinophilic Oesophagitis in North Denmark Region. Diagnostic delay is the time from first symptom to diagnosis. Second biopsy delay is the time from initial biopsy to second biopsy. Symptomatic remission delay is the time from initial treatment to symptomatic remission and histologic remission delay is the time from initial treatment to first oesophageal biopsy showing histologic remission (< 15 EOS/HPF)

The median diagnostic delay among children was four years and six months (Fig. 4A). The time to second biopsy was one year and one month (Fig. 4A). The median symptomatic remission delay and histologic remission delay was five years and six months, respectively (Fig. 4B).

### Treatment and follow-up

**Fig. 5:**
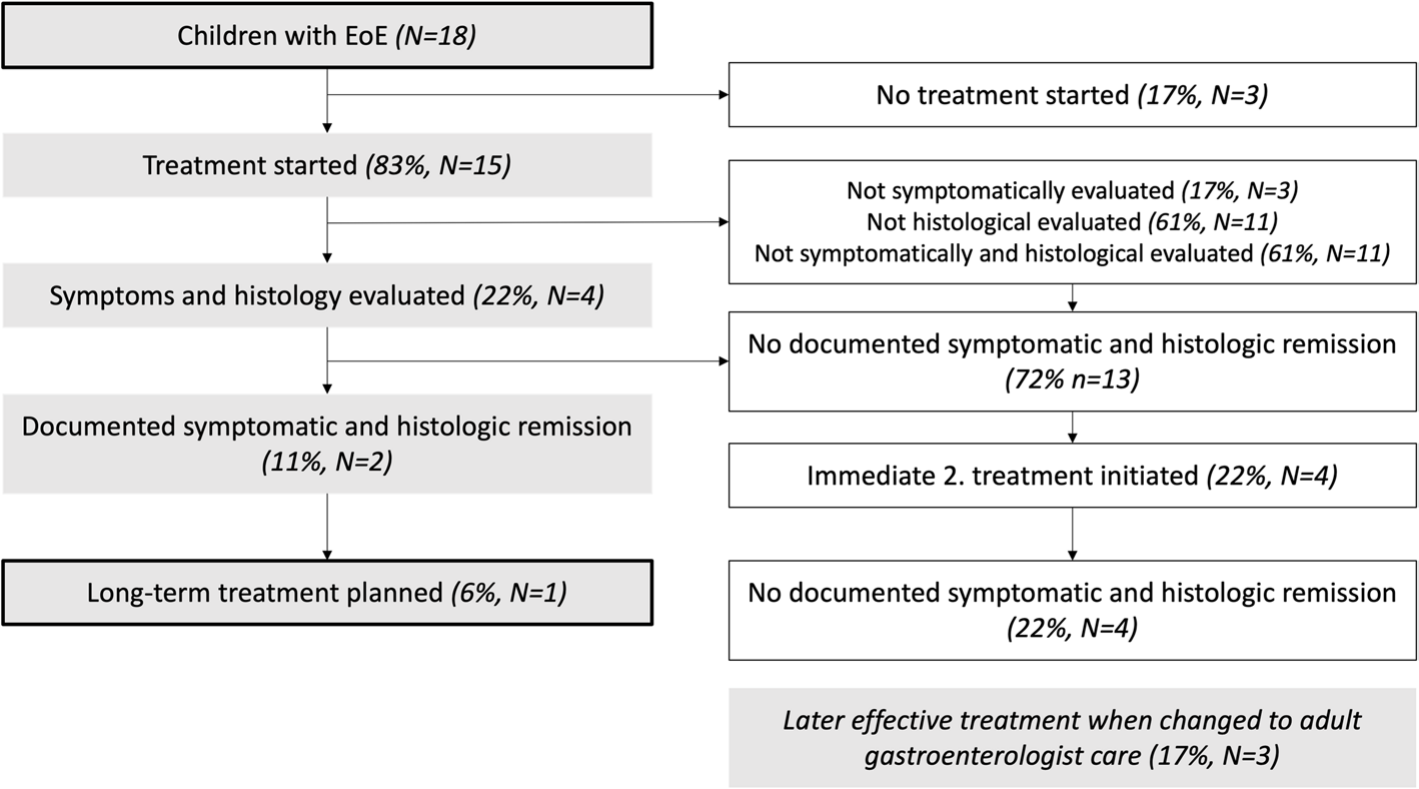
Initial treatment and follow-up of Eosinophilic Oesophagitis in children in North Denmark Region. Effective treatment is defined as achieving both symptomatic and histologic remission and having a long-term treatment plan for maintenance. Long-term treatment is defined as treatment that maintains remission. Abbreviations: EoE, Eosinophilic Oesophagitis; n, Number

Fifteen of the 18 children were offered EoE-specific treatment. Treatment consisted of proton-pump inhibitor (PPI) or dietary treatment and was initiated within 1–2 months following diagnosis. After initial treatment, four children were both symptomatically and histologically evaluated, and two had both symptomatic and histologic remission. Only one of these two children had a long-term treatment plan, defined as a plan that maintains remission. In the children where initial treatment failed to reach documented symptomatic and histologic remission, only four received immediate second EoE treatment. The immediate second treatment failed to reach documented symptomatic and histologic remission in all of the four children. Three children received effective treatment by gastroenterologists after transition to adult care at the age of 18 years. In total, only one of 18 children received effective treatment defined as documented complete remission and a long-term treatment plan (Fig. 5).

## Discussion

This retrospective register-based study of EoE in children in NDR in 2007–2017 based on the DanEoE cohort showed an incidence of 0.86/100,000 per year. The median diagnostic delay was four years and six months, and of the 15 children starting EoE treatment, one was effectively treated.

### Incidence

The incidence of EoE among children in the NDR in 2007–2017 was much lower than expected. The incidence in similar studies in Europe is more than six times higher [8]. The incidence among children in the only other study in Denmark was calculated in 2008 [10] and was two times higher than the incidence calculated in this study. As the incidence of EoE has been documented to increase in the entire western world, the low incidence found in the present study is most likely due to lack of detection. Our group found a 50-fold increase in EoE among adults in NDR in 2007–2017, following the introduction of a new biopsy protocol in 2011 [9]. Accoding to this protocol, all patients with dysphagia should have sampled at least eight biopsies regardless of macroscopic findings. This single intervention resulting in such a dramatic increase in incidence suggests that the awareness of EoE in NDR had been low. Moreover, the incidence of EoE reported in other European countries is very similar among adults and children [8]. The incidence found in this study was eight times lower in children compared to adults suggesting that not all children with EoE are diagnosed in NDR. Additionally, the male:female ratio of 17:1 and the lack of children aged 0–2 diagnosed with EoE in our study population differed from the literature. Spergel et al. 2009 included 620 children and found a male:female ratio of 3:1 [15], which is consistent with what has been reported in adults in NDR [16]. Furthermore, the results by Spergel et al. showed that 35% of children with EoE are diagnosed before the age of three and 68% before the age of 6 [15]. This suggests that many children with EoE aged 0–2 years are not found in NDR despite suffering from the disease [3, 15]. Regarding complications, food impaction was overrepresented in our study population compared to the literature [4]. This suggests that the children were diagnosed late, since food impaction is a late-stage manifestation of EoE [17]. We speculate that the diagnosis of EoE in these children was not considered until complications emerged, which may be explained by diffuse symptoms such as vomiting and abdominal pain being harder to attribute to the oesophagus and thus less likely to be associated with EoE.

### Diagnostic delay

The median diagnostic delay in our study was four years and six months among all children in our cohort. This was in concordance with other studies in similar populations in Europe [18, 19]. Diagnostic delay may have serious consequences, as untreated EoE over time can cause irreversible changes in the oesophagus [20]. The diagnostic delay may also indicate that knowledge of EoE among general practitioners and pediatricians is lacking. An explanation might be that national guidelines on EoE in children have yet to be implemented for pediatricians or general practitioners. In 2014, the first Danish review on EoE was published in the journal read by most general practitioners [21]. The only official guidelines for EoE in Denmark is from 2015 concerning adults. In addition to this, the current biopsy protocol for children in NDR recommends two biopsies from the distal part of the oesophagus and two biopsies from the middle or proximal part of the oesophagus, which fails to meet 100% sensitivity [3].

### Treatment and follow-up

Our results not only demonstrated a considerably prolonged diagnostic delay, but also suggests a problematic course of treatment. Of the 15 children that received treatment, twelve were symptomatically evaluated, four were histologically evaluated, and four were both symptomatically and histologically evaluated. It is not surprising that most of the treated children had undergone symptomatic evaluation. It is, however, surprising that only four children were histologically evaluated, as this is necessary to assess the effectiveness of the EoE-specific treatment. Additionally, twice as many children achieved symptomatic than histologic remission, which may indicate lack of attention to the underlying cause of the symptoms. This is problematic as oesophageal inflammation does not necessarily subside? when symptoms do. The lack of treatment may be explained by insufficient knowledge of the treatment guidelines for EoE. Lack of adhenrence to guidelines may explain the remission delay of almost 5 years. In our study, in the two children who achieved both histologic and symptomatic remission, only one of them had a long-term treatment plan. This is important as EoE will relapse without life-long treatment of the inflammation [8]. In summary, only one of the 18 children diagnosed with EoE in NDR from 2007–2017 achieved both symptomatic and histologic remission and had a long-term treatment plan.

### Strengths and limitations

The generalizability of the results of this study is strong due to the complete and valid data in the DanEoE database covering the entire population of NDR. The NDR comprising one tenth of the Danish population, is well defined geographically and the composition of people reflects the Danish population. However, the small study population limits the external validity. In small data sets, outliers pose difficulties because they affect the median values more than would be the case in larger samples. On the other hand, the fact that the study population is small further adds to arguments that the EoE diagnosis in children is gravely underdiagnosed in Denmark.

Only children from 2007–2017 with biopsies confirming EoE were included in this study. The calculated incidence would have been more accurate if the study included more recent data, which unfortunately was not possible.

Because not all data with relevance to determining the diagnostic delay was available, four children were excluded in the calculation of diagnostic delay, which may have altered our results.

## Conclusions

The calculated incidence among children in NDR with EoE was 0.86/100,000 children per year (2007–2017), which was lower compared with similar studies. The low incidence, poor remission rates and a lack of follow-up suggest that EoE is underdiagnosed and insufficiently treated among children in NDR. Our findings suggest that more knowledge of and attention to EoE in children could increase incidence, reduce diagnostic delay and result in more effective treatment.

## Data Availability

The datasets used and/or analysed during the current study are available from the corresponding author on reasonable request. Data is not shared up front, as they are part of a database in continuous research activity.

## Abbreviations

EoE: Eosinophilic oesophagitis
PPI: Proton pump inhibitor
NDR: North Denmark Region
EOS/HPF: Eosinophils per high-power field
